# Supplementary material for: Estimated glomerular filtration rate predicts 30-day mortality in medical emergency departments: Results of a prospective multi-national observational study
Source: PLoS One. 2020 Apr 6;15(4):e0230998. doi: 10.1371/journal.pone.0230998 (PMC7135226; doi:10.1371/journal.pone.0230998)
Supplement: S1 Table — (DOCX) [file pone.0230998.s001.docx]

**Table A1 Baseline characteristics of the total cohort by centers**

|  | **Clearwater Hospital** | **Hôpital de la Salpêtrière** | **Kantonsspital Aarau** |
| --- | --- | --- | --- |
| **Number (%)** | 969 | 1460 | 4554 |
| Age, median (IQR) | 69 (55, 81) | 55 (38, 69) | 63 (47, 76) |
| Male Sex | 487 (50.3%) | 718 (49.2%) | 2518 (55.3%) |
| **Vital signs, median (IQR)** |  |  |  |
| Systolic blood pressure, (mmHg) | 140 (121, 163) | 133 (119, 149) | 138 (122, 155) |
| Diastolic blood pressure, (mmHg) | 78 (67, 89) | 79 (70, 90) | 81 (71, 91) |
| Confusion | 38 (3.9%) | 29 (2.0%) | 450 (9.9%) |
| Pulse rate (bpm) | 82 (71, 98) | 86 (73, 99) | 82 (70, 95) |
| O2 Saturation (%) | 97 (95, 99) | 98 (96, 99) | 96 (94, 97) |
| Temperature (°C) | 36.5 (36.1, 36.8) | 36.7 (36.4, 37) | 36.9 (36.6, 37.4) |
| **Laboratory results, median (IQR)** |  |  |  |
| Hemoglobin (g/l), | 12.9 (11.4, 14.4) | 13.7 (12.4, 14.8) | 13.6 (12.2, 14.9) |
| Leukocyte count (G/l) | 8.3 (6.3, 10.9) | 8.1 (6.3, 10.8) | 8.45 (6.6, 11.0) |
| Glucose (mmol/l) | 138 (136, 140) | 138 (137, 140) | 139 (137, 141) |
| Creatinine (µmol/l) | 79.6 (70.7, 106.1) | 67.0 (55.5, 84.0) | 85.0 (70.0, 106.0) |
| eGFR (ml/min/1.73m^2^) | 69(50, 87) | 97 (77, 111) | 76 (55, 95) |
| CRP (mg/l) | 4.5 (.95, 11.25) | 4 (.9, 29) | 5.5 (0, 27.8) |
| PCT (µg/l) | 0.09 (0.06, 0.15) | 0.08 (0.06, 0.13) | 0.08 (0.06, 0.13) |
| Copeptin (pmol/l) | 14.9 (5.6, 59.7) | 7.8 (4.0, 19.4) | 11.6 (4.7, 43.8) |
| **Main diagnosis, number (%)** |  |  |  |
| Infection | 125 (12.9%) | 248 (17.0%) | 653 (14.3%) |
| Cardiovascular | 360 (37.2%) | 276 (18.9%) | 1000 (22.0%) |
| Metabolic | 39 (4.0%) | 89 (6.1%) | 62 (1.4%) |
| Cancer | 38 (3.9%) | 67 (4.6%) | 234 (5.1%) |
| Neurological | 157 (16.2%) | 248 (17.0%) | 1144 (25.1%) |
| Gastroinstestinal | 133 (13.7%) | 346 (23.7%) | 498 (10.9%) |
| Pulmonary | 58 (6.0%) | 65 (4.5%) | 169 (3.7%) |
| Other | 59 (6.1%) | 121 (8.3%) | 794 (17.4%) |
| **Comorbidities, number (%)** |  |  |  |
| Cancer | 79 (8.2%) | 206 (14.1%) | 675 (14.8%) |
| Renal failure | 75 (7.7%) | 97 (6.6%) | 692 (15.2%) |
| Congestive heart disease | 113 (11.7%) | 83 (5.7%) | 285 (6.3%) |
| COPD | 49 (5.1%) | 78 (5.3%) | 228 (5.0%) |
| Coronary heart disease | 105 (10.8%) | 172 (11.8%) | 557 (12.2%) |
| Dementia | 64 (6.6%) | 9 (0.6%) | 147 (3.2%) |
| Diabetes mellitus | 216 (22.3%) | 182 (12.5%) | 677 (14.9%) |
| History of Stroke | 5 (0.5%) | 104 (7.1%) | 455 (10.0%) |
| Hypertension | 465 (48.0%) | 391 (26.8%) | 1913 (42.0%) |
| **Inpatient treatment, number ,(%)** | 968 (100%) | 565 (38.7%) | 2571 (80.2%) |
