# Supplementary material for: Estimated glomerular filtration rate predicts 30-day mortality in medical emergency departments: Results of a prospective multi-national observational study
Source: PLoS One. 2020 Apr 6;15(4):e0230998. doi: 10.1371/journal.pone.0230998 (PMC7135226; doi:10.1371/journal.pone.0230998)
Supplement: S2 Table — (DOCX) [file pone.0230998.s002.docx]

**Table A2 Associations of eGFR with adverse clinical outcome in an univariate model and after adjustment for laboratory results**

|  |  | **eGFR (ml/min/1.73m^2^)** | | | | | |  |
| --- | --- | --- | --- | --- | --- | --- | --- | --- |
|  | **Total cohort** | **>90** | **60-89** | **45-59** | **30-44** | **15-29** | **<15** | **Continuous eGFR/10** |
| **30-day mortality, number (%)** | 325/6983 (4.65%) | 47/2544 (1.8%) | 88/2504 (3.5%) | 61/889 (6.9%) | 62/561 (11.1%) | 42/309 (13.6%) | 25/176 (14.2%) |  |
| Unadjusted model, OR (95% CI), p-value |  | Ref | 1.94 (1.35 to 2.77), p<0.001 | 3.91 (2.65 to 5.77), p<0.001 | 6.6 (4.46 to 9.76), p<0.001 | 8.36 (5.41 to 12.91), p<0.001 | 8.8 (5.27 to 14.68), p<0.001 | 1.28 (1.24 to 1.33), p<0.001 |
| adjusted for CRP |  | Ref | 1.94 (1.28 to 2.95), p=0.002 | 3.58 (2.28 to 5.62), p<0.001 | 5.97 (3.8 to 9.4), p<0.001 | 5.94 (3.55 to 9.93), p<0.001 | 6.57 (3.49 to 12.39), p<0.001 | 1.24 (1.19 to 1.3), p<0.001 |
| adjusted for Hb |  | Ref | 1.74 (1.21 to 2.5), p=0.003 | 3.11 (2.1 to 4.61), p<0.001 | 4.55 (3.05 to 6.81), p<0.001 | 4.74 (3 to 7.49), p<0.001 | 4.79 (2.81 to 8.17), p<0.001 | 1.2 (1.15 to 1.25), p<0.001 |
| adjusted for Copeptin |  | Ref | 1.36 (0.94 to 1.96), p=0.103 | 2 (1.33 to 3.01), p=0.001 | 2.56 (1.68 to 3.91), p<0.001 | 2.59 (1.61 to 4.14), p<0.001 | 2.21 (1.26 to 3.87), p=0.006 | 1.11 (1.07 to 1.16), p<0.001 |
| adjusted for CRP, Hb, Copeptin |  | Ref | 1.22 (0.79 to 1.88), p=0.365 | 1.56 (0.97 to 2.52), p=0.07 | 1.7 (1.03 to 2.8), p=0.038 | 1.17 (0.66 to 2.08), p=0.588 | 1.07 (0.53 to 2.15), p=0.858 | 1.02 (0.97 to 1.08), p=0.381 |
| Models were stepwise adjusted for age (model 1), age, and gender (model 2), age, gender,main diagnosis, and comorbidities (model 3)  ORs in eGFR/10 correlate to a decrease in GFR by 10ml/min/1.73m^2^ | | | | | |  |  |  |
|  |  |  |  |  |  |  |  |  |
