# Supplementary material for: Estimated glomerular filtration rate predicts 30-day mortality in medical emergency departments: Results of a prospective multi-national observational study
Source: PLoS One. 2020 Apr 6;15(4):e0230998. doi: 10.1371/journal.pone.0230998 (PMC7135226; doi:10.1371/journal.pone.0230998)
Supplement: S3 Table — (DOCX) [file pone.0230998.s003.docx]

**Table A3 Associations of eGFR with adverse clinical outcome in univariate and multivariate models without patients with a eGFR<15 ml/min/1.73m^2^**

|  |  | **eGFR (ml/min/1.73m^2^)** | | | | | |
| --- | --- | --- | --- | --- | --- | --- | --- |
|  | **Total cohort** | **>90** | **60-89** | **45-59** | **30-44** | **15-29** | **Continuous eGFR/10** |
| **30-day mortality, number (%)** | 325/6983 (4.65%) | 47/2544 (1.8%) | 88/2504 (3.5%) | 61/889 (6.9%) | 62/561 (11.1%) | 42/309 (13.6%) |  |
| Odds ratio (95% CI), p-value |  |  |  |  |  |  |  |
| Unadjusted Model |  |  | 1.94 (1.35 to 2.77), p<0.0010 | 3.91 (2.65 to 5.77), p<0.001 | 6.6 (4.46 to 9.76), p<0.001 | 8.36 (5.41 to 12.91), p<0.001 | 1.30 (1.25 to 1.36), p<0.001 |
| Model 1 |  | Ref | 0.77 (0.51 to 1.16), p=0.213 | 1.05 (0.66 to 1.68), p=0.833 | 1.7 (1.05 to 2.73),  p=0.03 | 2.12 (1.27 to 3.54), p=0.004 | 1.13 (1.06 to 1.19), p<0.001 |
| Model 2 |  | Ref | 0.76 (0.5 to 1.13),  p=0.175 | 1.04 (0.65 to 1.66), p=0.863 | 1.66 (1.03 to 2.67), p=0.037 | 2.09 (1.26 to 3.5),  p=0.005 | 1.12 (1.06 to 1.19), p<0.001 |
| Model 3 |  | Ref | 0.82 (0.55 to 1.24), p=0.357 | 1.25 (0.78 to 2),  p=0.363 | 1.82 (1.11 to 2.96), p=0.017 | 2.49 (1.46 to 4.25), p=0.001 | 1.13 (1.07 to 1.20), p<0.001 |
| Models were stepwise adjusted for age (model 1), age, and gender (model 2), age, gender, main diagnosis, and comorbidities (model 3)  ORs in eGFR/10 correlate to a decrease in GFR by 10ml/min/1.73m^2^ | | | | | | | |
