# Supplementary material for: Estimated glomerular filtration rate predicts 30-day mortality in medical emergency departments: Results of a prospective multi-national observational study
Source: PLoS One. 2020 Apr 6;15(4):e0230998. doi: 10.1371/journal.pone.0230998 (PMC7135226; doi:10.1371/journal.pone.0230998)
Supplement: Attachment — Submitted filename: Response to Reviewers.docx [file pone.0230998.s004.docx]

Mr. Tatsuo Shimosawa, M.D., Ph.D.
Academic Editor, PLOS ONE

Basel, the 5^th^ of November, 2019

Dear Doctor Shimosawa,

Thank you, for the opportunity to submit a revised manuscript of our study “Estimated glomerular filtration rate predicts 30-day mortality in medical emergency departments: results of a prospective multi-national observational study.” ID: PONE-D-19-23285) for publication in PLOS ONE. We appreciate the time invested in the manuscript by the editorial team and particularly the referees. The suggestions helped us to significantly improve the manuscript. The response to the question of the editorial team and the referees are listed in the point to point answer below.

We hope that the manuscript has reached publication quality for PLOS ONE.

Best regards

Laurent Haas, for all the co-authors

To Reviewer #1:

Dear Doctor Kawarazaki,

Thank you very much for the significant time, which you put into the evaluation of our manuscript. We appreciate your constructive comments, which helped us to improve the manuscript. We have incorporated your comments as follows.

Best regards

Laurent Haas.

*1) Primary and secondary outcomes were adjusted for age, gender, main diagnosis, and comorbidities. Why were laboratory data not adjusted for? It seems that those with lower eGFR had higher CRP, copeptin and lower Hb which are probably related to the outcomes.*

According to your suggestion we performed another analysis adjusting for the laboratory markers and added a table (Table A2) in the supplementary material.

When adjusted for selected variables (Hb, CRP, copeptin), the statistical significance of our main explanatory variable of interest, eGFR, becomes less strong. This suggests that copeptin and eGFR capture some of the same variation with respect to our outcome variable.

*2) The proportion and basic characteristics of patients recruited from the three tertiary care centers should be mentioned (perhaps as supplementary data).*

We have added the number of patients per country/center in the results section of the main manuscript (revised manuscript Page 8 paragraph 1).

Moreover, we have added patient characteristic stratified by center as supplementary data (Supplementary Table A4).

*3) Limitation of not having data on race should be mentioned since this will incorrectly calculate eGFR and race is also probably an important confounding factor of the outcome. Is data on race really not available at least for the cohort from the US?*

Race has of course a significant influence on eGFR, but this information was unfortunately not entered in the utilized database. We regret this limitation with regard to our data. We have added a note of caution and further discussed that in section of the limitation of the study (revised manuscript page 18 paragraph 2).

*4) I am not really convinced that this study states a novel insight because serum Cr or eGFR has already been shown to be a predictive factor in chronic and acute settings. Mixing the chronic and acute setting will probably yield thee same results. Can you be more specific on how the result or method of this study is new?*

Thank you for this important comment. As outlined in the introduction of our manuscript multiple studies assessing the predictive power of SCr and eGFR in a chronic setting. We are not aware of any larger studies of emergency patients. Given the necessity for a fast and easy (cost-efficient) biomarker for risk stratification in an emergency setting, we believe that it is worthwhile to assess its aptitude with respect to this setting. But it is correct that for the diseases already studied, our data is confirmative in nature. This was discussed in more detail (page 4 paragraph 3).

Reviewer #2:

Dear Doctor Kent Doi,

We appreciate your time and effort which you put into the evaluation of our manuscript. We think that these helped us to significantly improve the revised manuscripted. We have incorporated your comments as follows.

Best regards

Laurent Haas.

*1) Were end-stage renal disease patients treated by maintenance dialysis included? If so, their eGFR should be different between before and after dialysis. This will cause a serious bias.*

As rightly suggested chronic hemodialysis HD patients have highly different creatinine values before and after dialysis, and therefore a high variance in eGFR. Hemodialysis patients were included in our study although this variable was not surveyed, so our models could not be adjusted for this circumstance. The exact proportion of this patients in the cohort is unknown. We assume that although it is higher than in the general population, the absolute quantity should be relatively low. Dialysis patients have an increased mortality rate. This should also be reflected in the data with high baseline creatinine (even after dialysis) compared to the general population. To address your comment we have added a table (Table A3) with our calculations excluding patients with Cat 5 patients (as this group contains patients on dialysis) in the supplementary materials. These results are described in in the revised manuscript on page 16 paragraph 4). The results remain robust after excluding Cat 5 patients.

*2) Cat 4 (eGFR 15-29) showed worse survival curve just before day 30 compared with Cat 5 (eGFR<15) (Figure 2). The reason for this should be investigated. Did Cat 5 include many dialysis patients?*

This is indeed a curious finding, as the Cat. 4 patients seem to have a higher in hospital mortality but a lower 30-day mortality. We added the following paragraph in our manuscript (Page 16, Paragraph 3) Cat.5. patients have a higher ICU admission rate. These patients could be perceived as “sicker” and more short-term medical resources might be attributed (as reflected by ICU admission) to them, influencing discharge and transfer practices. Experts are discussing in-hospital vs 30-day mortality for outcome prediction/quality assessment and 30-day mortality may be a more valid measure, since it is a fixed point in time. In-hospital mortality is primary influenced by hospital transfer policies and long term (30, 60, 90 days) is mainly determined by diagnosis [1, 2]. Mortality seems to “catch up” with the Cat.5 patients on day 30.

The number of hemodialysis patients has been addressed under the first question.

*3) It is a little bit surprising that most (72.8%) pts were treated as inpatients in this ED cohort. Can the severity of the enrolled patients be evaluated? Can qSOFA score be calculated?*

We now provide frequency of inpatient care in the newly added Table A2 in the supplementary data (please see also our answer to question 2 of Reviewer #1). As you can see, there was a broad difference in frequency of inpatient care in the participating hospitals. This can be explained by the different health care systems studied. In the US-hospital and in the Swiss hospital medical patients are admitted through the emergency department for inpatient care, and patients with high probability of outpatient care are seen in a separate department (outpatient emergency service). In the French hospital, all of the patients are seen in the same department, which explains the distinct difference in inpatient treatment.

Unfortunately, data for respiratory rate is limited. That’s why we are not able to calculate SOFA score in a meaningful number of patients.

*4) As mentioned in the limitation section, this study did not distinguish AKI from CKD. However, because 72% of the enrolled patients admitted, subanalysis regarding AKI, CKD, acute-on-chronic can be performed when limited to the inpatients. This analysis will provide more precise role of single measurement of eGFR at ED.*

Indeed, most patients were treated as inpatients. In the database, there is only a one-time creatinine measurement available to us. During hospitalization creatinine monitoring was certainly performed but it was not integrated in the database, as the initial study design was to analyze the predictive power of novel biomarkers during triage in the emergency department. A follow up creatinine would have been interesting to differentiate between AKI and CKD. This would have provided additional information to the data and value to the paper.

We are aware of the limitations of the data; however, we believe that our data provide interesting information due to its large sample size and real-life cohort. It is a good reflection of the diverse patient population encountered in daily practice. We have emphasized this limitation in the revised manuscript (page 17 paragraph 3).

References:

1.	van Rijn M, Buurman BM, Macneil Vroomen JL, Suijker JJ, ter Riet G, Moll van Charante EP, et al. Changes in the in-hospital mortality and 30-day post-discharge mortality in acutely admitted older patients: retrospective observational study. Age and Ageing. 2016;45(1):41-7. doi: 10.1093/ageing/afv165.

2.	Vasilevskis EE, Kuzniewicz MW, Dean ML, Clay T, Vittinghoff E, Rennie DJ, et al. Relationship between discharge practices and intensive care unit in-hospital mortality performance: evidence of a discharge bias. Med Care. 2009;47(7):803-12. Epub 2009/06/19. doi: 10.1097/MLR.0b013e3181a39454. PubMed PMID: 19536006.
