# Supplementary material for: Estimated glomerular filtration rate predicts 30-day mortality in medical emergency departments: Results of a prospective multi-national observational study
Source: PLoS One. 2020 Apr 6;15(4):e0230998. doi: 10.1371/journal.pone.0230998 (PMC7135226; doi:10.1371/journal.pone.0230998)
Supplement: Attachment — Submitted filename: Response to Reviewers.docx [file pone.0230998.s005.docx]

Mr. Tatsuo Shimosawa, M.D., Ph.D.
Academic Editor, PLOS ONE

Basel, the 27^th^ of February, 2020

Dear Doctor Shimosawa,

Thank you, for the opportunity to submit a second revision of our manuscript “Estimated glomerular filtration rate predicts 30-day mortality in medical emergency departments: results of a prospective multi-national observational study.” ID: PONE-D-19-23285) for publication in PLOS ONE. We appreciate the time invested by the editorial team and the referees.

We appreciate the positive review of the first referee and addressed his minor comment as suggested. The second review was negative with the “*Unfortunately, the authors could not add any data because of data availability of their database. That suggests this study has numbers of significant limitations.”*

The limitations remain, as we are limited to the information of the database. We decided to address the negative comment in the following way.

We rewrote large parts of the study to better described the focus of the manuscript. We only have cross-sectional creatine at the time of admission, and this was the scope of study.

2. For the two comments in which the referee asked for further information which were not included in the available dataset we tried to further evaluate the potential bias and add information which might help to answer the question of the referee.

3. The text was shortened and focused on the available information.

We hope that with these changes it has reached publication quality for PLOS ONE.

Best regards

Laurent Haas, for all the co-authors

To Reviewer #1:

Dear Doctor Kawarazaki,

Thank you very much for your comment (as addressed below, and your positive evaluation. We really appreciate your time invested.

Best regards

Laurent Haas

*1) Just one minor comment. Table A4 has systolic BP and diastolic BP the other way round. All comments have been addressed.*

Thank you for your perceptiveness. We have corrected the mistake and also some minor formatting issues in our tables.

Reviewer #2:

Dear Doctor Kent Doi,

Thank you for your work on our manuscript. In your comment you pointed to the limitations of the study and particularly the missing information. With this in mind, we returned to your comments of the first review. In this second revision we further addressed your comments as follows.

*1) Were end-stage renal disease patients treated by maintenance dialysis included? If so, their eGFR should be different between before and after dialysis. This will cause a serious bias.*

The text was extensively revised. Excluding patients with an eGFR below 15 ml/min (Supplemental Material Table A3) did not change the results significantly. The limitation was included in the Abstract and Discussion, respectively.

*2) Cat 4 (eGFR 15-29) showed worse survival curve just before day 30 compared with Cat 5 (eGFR<15) (Figure 2). The reason for this should be investigated. Did Cat 5 include many dialysis patients?*

This remains as an intriguing finding. The number of patients and the event rate is small in patients with a eGFR<15 ml/min. Only three events would have lead to the same event rate as in the group with a eGFR 15-30 ml/min. Therefore, the group size might be of importance. Furthermore, 30-day mortality might be the better parameter as discussed.

*3) It is a little bit surprising that most (72.8%) pts were treated as inpatients in this ED cohort. Can the severity of the enrolled patients be evaluated? Can qSOFA score be calculated?*

Our ED consist of two wards. One is managed by general physicians and foreseen for patients likely to be treated as outpatients. In the other ward, patients likely to be treated as inpatients are assigned to. The study enrolled only patients in the latter ward, which explains the high rate of inpatients.

qSOFA cannot be calculated as the information is not available. The qSOFA would describe the severity in septic patients. Only, 15% suffered from infectious diseases. Therefore, the gained information would be limited.

*4) As mentioned in the limitation section, this study did not distinguish AKI from CKD. However, because 72% of the enrolled patients admitted, subanalysis regarding AKI, CKD, acute-on-chronic can be performed when limited to the inpatients. This analysis will provide more precise role of single measurement of eGFR at ED.*

Based on the helpful suggestions of the reviewers, the text was extensively revised and focused on the answers which can be given with the current dataset.
